# Spontaneous Poisonous Changes in Meat and Bread

**Published:** 1833

**Authors:** 


3. *Spontaneous Poisonous Changes in Bread and Meat.*—M. Chevallier has published a paper in the *Journ. de Chem. Med.* on certain changes which bread and meat, long kept, are apt to undergo, by which they are rendered poisonous. The following case occurred in the practice of M. Bricheteau, in August, 1831. A woman, aged about 40, purchased of a pork butcher and eat through the day, some slices of bacon. In the evening M. Bricheteau found that she had been vomiting for several hours.

"The abdomen was excessively tender. She had frequent stools, with tenesmus, and she complained of general pain. Cataplasms were applied to the abdomen, and she was ordered lavements, diluent drinks, and absolute diet. Notwithstanding this treatment, the patient had that night above fifty stools, and the abdominal pains continued very severe. Leeches were consequently applied, a warm bath ordered, and the previous treatment continued. In two days the patient recovered. These symptoms at such a time might have readily been attributed to other causes, had not a young woman, who had eaten a very small morsel of the same meat, experienced analagous accidents. And it further appeared that a third person had been very ill after eating pork purchased at the same time and place.

On the 30th of May, 1832, we were directed to institute an official inquiry re pecting an occurrence of the same nature, and which gave rise to the subjoined report:"

In May, 1832, a similar accident happened to another family, apparently from a like cause. A commission, consisting of MM. Durocher, Geeury and Chevallier, was directed to inquire into the circumstances of the case. The following is the result of their inquiry:

"*Examination of the Meat.*—The meat, a part of which had occasioned the illness of the female, was composed of several pieces cut from a lump of a preparation known in the pork trade by the name of *Italian cheese*, made of mixed fragments, strongly seasoned, and converted into a kind of compact pie, which is sold in slices. The pieces we examined were covered, some with blue, and others with green mould, the latter circumstance occasioning a copery appearance. Having divided a portion into three parts, one was treated with distilled water, and the solution tested by reagents, which proved the absence of any poisonous metal. Another part was treated with distilled water acidulated with nitric acid; the solution thus obtained was evaporated, the residuum redissolved in water, and tested by reagents, which, as before, gave no indication of any known poison. The last part of the meat was introduced into a new crucible, carbonized and incinerated. The ashes did not contain the least traces of copper. The same experiments repeated on the meat found at the shop of the Sieur L., were attended with the same negative results. From these facts it follows that the meat in question contained no copper, but that it had undergone a marked alteration capable of producing the accidents in question; nor is this the first example of poisoning by this particular substance. Dr. Paulus, of Saltz, has already related the history of seven persons who became violently ill after eating *Italian cheese*, and of whom three died. In 1824, a family named Plagneard, at Paris, were also very dangerously affected after partakinn of a ham pie which contained no metallic poison, but in which the alteration in question had commenced."

In the London Lancet, is contained an account of a case of poisoning by mouldy bread. It occurred in the family of the beadle of the parish of Hammersmith, Eng.

"His wife purchased in the morning a loaf of bread, of which she ate a slice at breakfast. Her son, 20 years of age, ate two slices of the same bread toasted; almost immediately after the meal, both became unwell, and diarrhœa, vomiting, and tenderness of the abdomen supervened, and several hours elapsed before these symptoms abated. The loaf, a considerable portion of which we obtained, was of yellowish color, though baked that morning, and heated for the ordinary length of time. It was sprinkled over with minute fungiform vegetations, the greater number of which were black, a few green, and several yellow. It was soft, wet, inelastic, and so tough that it could be drawn into strings. Its taste was unpleasant, its smell acrid, and it reddened litmus paper when laid upon it."

The mould, collected and eaten separately, was found to be innoxious. It was ascertained by various experiments, that no mineral poison was present.

"On analysis of the bread, it was found to contain the due proportion of starch, amidine, sugar, and earthy substances, but the gluten had undergone a marked alteration in its proportions."

